# Novel eicosanoid signature in plasma provides diagnostic for metabolic dysfunction-associated steatotic liver disease

**DOI:** 10.1016/j.jlr.2024.100647

**Published:** 2024-09-18

**Authors:** Oswald Quehenberger, Aaron M. Armando, Tiffany H. Cedeno, Rohit Loomba, Arun J. Sanyal, Edward A. Dennis

**Affiliations:** 1 Department of Pharmacology, University of California San Diego, La Jolla, CA, USA; 2 MASLD Research Center, Division of Gastroenterology and Hepatology, Department of Medicine, University of California San Diego, La Jolla, CA, USA; 3 Division of Gastroenterology, Department of Internal Medicine, Virginia Commonwealth University School of Medicine, Richmond, VA, USA; 4 Department of Chemistry and Biochemistry, University of California San Diego, La Jolla, CA, USA

**Keywords:** Lipidomics, MASLD, MASH, NAFLD, NAFL, NASH, fatty liver disease, eicosanoids, inflammation, arachidonic acid metabolism

## Abstract

There is a clinical need for a simple test implementable at the primary point of care to identify individuals with metabolic dysfunction-associated steatotic liver disease (MASLD) in the population. Blood plasma samples from adult patients with varying phenotypes of MASLD were used to identify a minimal set of lipid analytes reflective of underlying histologically confirmed MASLD. Samples were obtained from the NIDDK Nonalcoholic Steatohepatitis Clinical Research Network (NASH CRN) NAFLD Database prospective cohort study (MASLD group; N = 301). Samples of control subjects were obtained from cohort studies at the University of California San Diego (control group; N = 48). Plasma samples were utilized for targeted quantitation of circulating eicosanoids, related bioactive metabolites, and polyunsaturated fatty acids by ultra-high performance liquid chromatography-mass spectrometry (UPLC-MS) lipidomics analysis. Bioinformatic approaches were used to discover a panel of bioactive lipids that can be used as a diagnostic tool to identify MASLD. The final panel of fifteen lipid metabolites consists of 12 eicosanoid metabolites and 3 free fatty acids that were identified to be predictive for MASLD by multivariate area under the receiver operating characteristics curve (AUROC) analysis. The panel was highly predictive for MASLD with an AUROC of 0.999 (95% CI = 0.986–1.0) with only one control misclassified. A validation study confirmed the resulting MASLD LIPIDOMICS SCORE, which may require a larger-scale prospective study to optimize. This predictive model should guide the development of a non-invasive “point-of-care” test to identify MASLD patients requiring further evaluation for the presence of metabolic dysfunction-associated steatohepatitis.

According to recent data from the National Health and Nutrition Examination Survey (NHANES), the prevalence of adult obesity in the United States significantly increased to 42.8% in 2018 (1, 2). Including less severe forms of obesity, approximately two-thirds of the adult general population is either overweight or obese. This has been associated with numerous adverse health consequences including the development of cardiovascular disease, type two diabetes mellitus, and several cancers (3, 4, 5, 6). Another key outcome of excess adiposity and insulin resistance is the development of metabolic dysfunction-associated steatotic liver disease (MASLD), which has increased globally to greater than 30% of the adult population (7). Of note, this condition was until recently referred to as nonalcoholic fatty liver disease (NAFLD). The two histological phenotypes of NAFLD, that is, a nonalcoholic fatty liver (NAFL) and nonalcoholic steatohepatitis (NASH) are now referred to as metabolic dysfunction associated steatotic liver (MASL) and metabolic dysfunction associated steatohepatitis (MASH) respectively (8, 9, 10, 11, 12). MASH tends to progress to cirrhosis more frequently than MAFL and should therefore be especially targeted for therapeutic intervention. The current approach for clinical evaluation of MASLD begins with an assessment of a FIB-4 (Fibrosis-4) score followed by additional testing including transient elastography in those with clinical risk factors (13, 14). FIB-4 is a commonly used laboratory aid based on age, AST (aspartate aminotransferase), ALT (alanine aminotransferase), and platelet counts (15); it was developed to evaluate the presence of underlying advanced fibrosis and its use in the context of MASLD assessment is also for fibrosis assessment. This does not however provide any insight into the presence of steatosis, the hallmark of MASLD. Confirmation of steatotic liver disease requires liver biopsy and either measurement of the continuous attenuation parameter by transient elastography or MRI-based methods (16, 17, 18, 19). A liver biopsy is an invasive procedure with potentially serious side effects. Transient elastography or MRI is not always widely available and is expensive, creating a barrier to the assessment of MASLD, which starts with the assessment of clinical risk factors (20, 21).

Obesity is the most common risk factor for MASLD (21, 22, 23). While 75% of the general population is overweight or obese, only 30%–40% of the general population have MASLD. Also, it has been reported that up to 20% of individuals with MASLD are lean and would thus be missed by current practice guidelines (24, 25). There is, therefore, a need for a diagnostic test that can be used in routine clinical settings to identify who has MASLD so that appropriate secondary tests to assess disease activity and fibrosis can be performed for risk stratification and clinical decision-making.

Lipidomic analysis of plasma provides a “snapshot” of the state of lipid metabolism in the body (26). The development of MASLD is closely linked to metabolic syndrome and altered systemic metabolism including delivery of a lipotoxic load of fatty acids to the liver, leading to inflammation, another hallmark of MASLD (27, 28). Free, unesterified polyunsaturated fatty acids such as linoleic acid, linolenic acid, arachidonic acid (AA), adrenic acid, eicosapentaenoic acid (EPA), and docosahexaenoic acid (DHA) can undergo enzymatic or non-enzymatic oxidation and other modifications to give rise to prostaglandins, leukotrienes and various other forms of oxygenated metabolites. These lipid metabolites, including their precursor fatty acids, are collectively referred to as “eicosanoids” (29). This class of lipids is highly bioactive and can also be secreted from the liver into the circulation actively via lipoproteins or extracellular vesicles (exosomes) or through vascular leakage. We consequently hypothesized that examination of the circulating eicosanoids would provide a diagnostic signature reflective of the underlying presence of MASLD. The study reported herein was aimed at identifying and validating a diagnostic eicosanoid-derived signature of MASLD, the results of which are reported herein.

## Materials and Methods

### Study population

All human plasma samples were collected as part of clinical studies and/or trials that abided by the policies of the institutional review boards of the institution responsible for the samples and NASH CRN protocols and were supplied for analysis as de-identified samples. The work abides by the Declaration of Helsinki principles. Studies were conducted under the following institutionally approved protocols of University of California, San Diego IRB00002758, Virginia Commonwealth University IRBHM20004644, and Johns Hopkins School of Public Health IRB00006382.

A retrospective-prospective analysis of plasma samples from adult patients with varying phenotypes of MASLD (n = 301) was performed and compared to controls (n = 48). The MASLD population was derived from the NIDDK NASH Clinical Research Network study cohort. It included samples from individuals who participated in the non-interventional DB1 and DB2 registry (NCT01030484) and also baseline samples from individuals who participated in the PIVENS trial (NCT00063622) and the FLINT trial (NCT01265498). For the hypothesis-building study, baseline samples from patients enrolled in these trials and who did not undergo further treatment were used. For the Validation Study, baseline samples from the FLINT trial were used.

Plasma samples were obtained within 90 days of an evaluable liver biopsy, which was performed to confirm MASLD. The liver histology was evaluated in a masked manner by the pathology committee of the NASH CRN using a validated protocol and the presence of MASLD and its individual histological features was documented using the NASH CRN histological classification system (30). The histological spectrum extended from steatosis alone to steatohepatitis with varying stages of fibrosis.

Blood samples were obtained in all cases in a fasted state followed by plasma separation, aliquoting, and freezing within 2–3 h using a pre-specified protocol. Samples were frozen and stored at −70°C at individual clinical centers and then transferred on dry ice to the NIDDK biorepository. Samples were transmitted from the biorepository to the laboratory for analysis on dry ice. Thus, there were no instances of freeze-thaw prior to the analysis of the samples for the current study.

Controls were collected by similar procedures and were defined by a normal clinical examination, normal liver enzymes and functions, and a magnetic resonance imaging-proton density fat fraction (MRI-PDFF) assessed liver fat content <5% and magnetic resonance elastography (MRE) assessed liver stiffness < 2.5% based upon previously published thresholds (31, 32). They were identified at a single center (UCSD) and characterized for purposes of this analysis (Table 1). For the Validation Study, a separate set of control samples were collected at a later time and selected by the same criteria.

**Table 1:** Patient characteristics by MASLD status (N = 349)

	Mean (±SD) or N (%)		*P*^a^
	Control (N = 48)	MASLD Patients (N = 301)	
Age (years)	52.3 (±14.5)	51.4 (±11.4)	0.3
Age			0.3
18-34	7 (15%)	31 (10%)	
35-54	18 (38%)	145 (48%)	
55-74	23 (48%)	125 (42%)	
Sex, male	22 (46%)	102 (34%)	0.08
Race			<0.001
Non-hispanic white	14 (29%)	234 (78%)	
Non-hispanic black	4 (8%)	6 (2%)	
Hispanic	25 (52%)	33 (11%)	
Other	5 (10%)	28 (9%)	
BMI (kg/m^2^)	30.1 (±4.1)	34.6 (±6.3)	<0.001
BMI category			<0.001
Underweight	0 (0%)	0 (0%)	
Normal	0 (0%)	9 (3%	
Overweight	26 (54%)	58 (19%)	
Obese	22 (46%)	233 (78%)	
Type 2 diabetes	10 (21%)	144 (48%)	<0.001
Total cholesterol (mg/dl)	181 (±39)	187 (±43)	0.4
Triglycerides (mg/dl)	105 (±42)	182 (±210)	0.01
LDL (mg/dl)	107 (±33)	111 (±38)	0.5
HDL(mg/dl)	53 (±15)	44 (±12)	<0.001
Bilirubin, total (mg/dl)	0.5 (±0.2)	0.6 (±0.3)	0.02
Aspartate aminotransferase, AST (U/L)	19 (±5)	57 (±37)	<0.001
Alanine aminotransferase, ALT (U/L)	17 (±5)	77 (±52)	<0.001
Alkaline phosphatase, ALP (U/L)	81 (±29)	82 (±27)	0.8
Fibrosis stage^b^			
0. None	48 (100%)	44 (15%)	
1a. Mild, zone 3 perisinusoidal		31 (10%)	
1b. Moderate, zone 3, perisinusoidal		40 (13%)	
1c. Portal/periportal only		8 (3%)	
2. Zone 3 and periportal, any combination		73 (24%)	
3. Bridging		84 (28%)	
4. Cirrhosis		21 (7%)	
MASH stage^b^			
Not MASLD	48 (100%)	0 (0%)	
0. MASLD, not MASH	0 (0%)	34 (11%)	
1a. borderline MASH, zone 3 pattern	0 (0%)	43 (14%)	
1b. borderline MASH, zone 1 periportal pattern	0 (0%)	1 (<1%)	
2. Definite MASH	0 (0%)	223 (74%)	
Time difference between lab exam and biopsy (day)^c^		45 (±118)	

a *P*-value from student *t* test for continuous variables and Fisher’s exact test for categorical variables.

b Biopsy not done for controls.

c Date of lab exam – date of biopsy.

### Chemicals and standards

All solvents were ultra-performance LC (UPLC) grade or better and were purchased from Thermo Fisher Scientific. All primary standards (PSTDs) for standard curves (136 individual standards) and deuterated internal standards (ISTDs) (18 deuterated standards) for eicosanoid (EIC) analysis were purchased from Cayman Chemicals (Ann Arbor, MI) or Enzo Life Sciences.

### Lipidomic analysis of eicosanoids

Eicosanoids were analyzed by UPLC-MS as previously described (33, 34). For isolation, aliquots of 50 ul plasma samples were diluted to 900 ul with PBS and spiked with a mixture of 18 deuterated ISTDs in 100 ul of ethanol. The eicosanoids were extracted using Strata-X reversed-phase SPE columns (8B-S100-UBJ, Phenomenex). Columns were activated with 3 ml of 100% methanol and then equilibrated with 3 ml of water containing 10% ethanol. After loading the samples, the columns were washed with 10% methanol to remove impurities, and the metabolites were then eluted with 1 ml of 100% methanol and stored at −80°C to prevent metabolite degradation. Prior to analysis, the eluent was dried under vacuum and re-dissolved in 50 ul of the UPLC solvent A (water/acetonitrile/acetic acid (60:40:0.02; v/v/v)) for UPLC/MS/MS analysis.

The separation of individual metabolites was performed on an Acquity UPLC system (Waters), equipped with a C18 BEH shield column (2.1 × 100 nm; 1.7 um; Waters), as described previously (34). Briefly, 10 ul of purified samples were injected and separated using a binary buffer system consisting of buffer A (described above) and buffer B composed of acetonitrile/2-propanol (50/50, v/v). At a flow rate of 0.5 ml/min, buffer A was held at 100% for 1 min followed by a gradient over 3 min to 55% buffer B, then further increased over 1.5 min to 100% buffer B and kept at this level for 0.5 min. The starting conditions were reconstituted in 1 min. The column was kept at 40°C and the samples at 4°C.

The eluting metabolites were analyzed by mass spectrometry (MS). For data collection, the UPLC was interfaced with a Sciex 6500 QTRAP hybrid triple quadrupole mass spectrometer (SCIEX). The instrument was operated in the negative ionization mode using a scheduled multiple reaction monitoring (MRM) method. The source settings were as follows: curtain gas (CUR = 20 psi), nebulizer gas (GS1 = 30 psi), turbo heater gas (GS2 = 20 psi), electrospray voltage (TEM = −4,500 V), source temperature 500°C, and collision gas (CAD = medium).

All eicosanoids were quantified by the stable isotope dilution method. Briefly, identical amounts of ISTDs were added to each sample and to all the PSTDs. Nine-point standard curves were generated for each of the 136 PSTDs, ranging from 0.03 ng to 10 ng. The linearity of the instrumental response goes over several orders of magnitude and fully covers the concentration range of the metabolites used to generate the calibration curve. To increase the accuracy of the model, we use the weighted least-square regression. Generally, the R-squared values for the standard curves of the individual eicosanoids are greater than 0.99. To calculate the amount of each eicosanoid in a sample, ratios of peak areas between endogenous eicosanoids and matching deuterated internal eicosanoids were calculated. Ratios were converted to absolute amounts by linear regression analysis of the standard curves. Currently, we quantify most eicosanoids at low femtomole levels.

To avoid to the extent possible any degradation of eicosanoids or non-enzymatic oxidation of PUFAs during sample preparation, the samples were thawed only once and all preparations were performed immediately on ice. The performance of the method was extensively tested in a previous study (34). For this purpose, pure standards were subjected to the extraction procedure or used directly for UPLC-MS analysis. We did not observe any significant differences in eicosanoid recovery and concluded that eicosanoids were not degraded or formed during the analytical process. Furthermore, we have analyzed a set of plasma quality control samples over a period of 3 years. We periodically thawed a sample for analysis and found that prolonged storage over 3 years at −80°C with a single thaw cycle did not significantly affect the integrity of the sample.

### Statistical analysis and biomarker identification

In all, 77 analytes were detectable in any of the control or patient samples. Of these, we removed any metabolite that was present in less than 80% of the samples. We rationalized that if metabolites are present in less than 80% of the patient samples, they could not be used meaningfully in clinical practice. Any non-detectable values were replaced with one-fifth of the minimum value for each analyte. The remaining 28 eicosanoids and PUFAs were used for data processing. Statistical analyses were performed by MetaboAnalyst 5.0 (35). The peak area of each metabolite was used without normalization and associations of the lipidomics features with the patient phenotype were determined by partial least squares discriminant analysis (PLS-DA). To determine the significance of the differences between the control and patient groups, *t* test analysis was performed. For identifying potential biomarkers and to evaluate their performances, multivariate area under receiver operating characteristics curve (AUROC) analysis based on the Random Forest for classification and univariate AUROC for feature ranking was performed.

## Results

### Patient characteristics

A total of 349 individuals including 301 with MASLD and 48 controls without MASLD were studied (Table 1). The ages of participants with MASLD were not statistically different than those of the controls (mean ages 52.3 vs. 51.4 years (*P* < 0.3). The distribution of ages in the two groups was also not statistically significantly different. Although as expected, the controls had a lower BMI (30.1 vs. 34.6 kg/m^2^, *P* < 0.001), 46% of the controls were obese. In contrast, 78% of MASLD subjects were obese. The prevalence of type two diabetes was also higher in those with MASLD (48% vs. 21%, *P* < 0.001), as expected.

Controls had normal liver enzymes and hepatic synthetic functions that were significantly different from the patients with MASLD (*P* < 0.001 for AST and ALT, *P* < 0.02 bilirubin). Amongst those with MASLD, 34 had steatosis while 44 had borderline steatohepatitis and 223 had definite steatohepatitis. About two-thirds of patients with MASLD had some degree of fibrosis (as noted in Table 1); the fibrosis stages were relatively evenly distributed between stages with 26%, 24%, 28% and 7% spread through stages 1,2,3 and 4, respectively.

### Eicosanoid profile in plasma of MASLD

A comprehensive analysis of circulating eicosanoids was performed in plasma from controls and MASLD patients. In all, 77 eicosanoid metabolites were detectable in at least one of the samples and the distribution of their concentration in controls and in those with MASLD was calculated. Several of these metabolites were present in the plasma only at low levels and their presence in circulation was inconsistent. To increase the reliability of the individual metabolites to distinguish between controls and MASLD, we only included eicosanoids that were present in at least 80% of the patients. We rationalized that if metabolites are present in less than 80% of the patient samples, they could not be used meaningfully in clinical practice. This stringency decreased the dataset to 32 analytes, which were used for all subsequent statistical analyses (Supplemental Table S1).

**Fig. 1:**
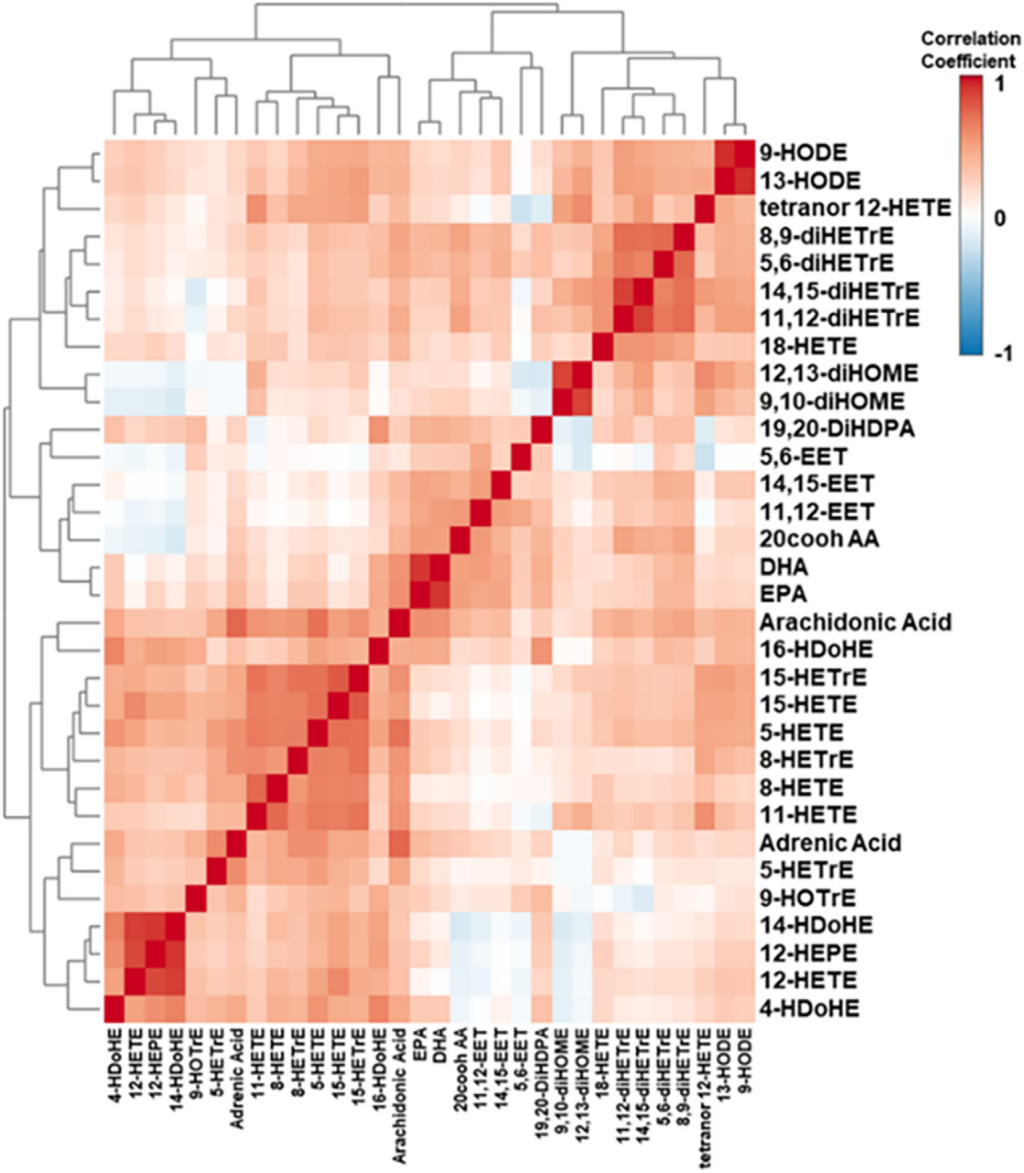
Correlation heat map of eicosanoids in control and MASLD plasma. Similarities between variables detected in the MASLD dataset were calculated using Spearman’s correlation. Shorter distances in the dendrograms indicate stronger relationships between the variables. Dark red shows a positive correlation between variables; dark blue shows a negative correlation between the variables. Abbreviations: 9-hydroxy-10E,12Z-octadecadienoic acid (9-HODE), 13-hydroxy-9Z,11E-octadecadienoic acid (13-HODE), 8-hydroxy-4Z,6E,10Z-hexadecatrienoic acid (tetranor 12-HETE), 8,9-dihydroxy-5Z,11Z,14Z-eicosatrienoic acid (8,9-diHETrE), 5,6-dihydroxy-8Z,11Z,14Z-eicosatrienoic acid (5,6-diHETrE), 14,15-dihydroxy-5Z,8Z,11Z-eicosatrienoic acid (14,15-diHETrE), 11,12-dihydroxy-5Z,8Z,14Z-eicosatrienoic acid (11,12-diHETrE), 18-hydroxy-5Z,8Z,11Z,14Z,16E-eicosapentaenoic acid (18-HETE), 12,13-dihydroxy-9Z-octadecenoic acid (12,13-diHOME), 9,10-dihydroxy-12Z-octadecenoic acid (9,10-diHOME), 19,20-dihydroxy-4Z,7Z,10Z,13Z,16Z-docosapentaenoic acid (19,20-diHDPA), 5,6-epoxy-8Z,11Z,14Z-eicosatrienoic acid (5,6-EET), 14,15-epoxy-5Z,8Z,11Z-eicosatrienoic acid (14,15-EET), 11,12-epoxy-5Z,8Z,14Z-eicosatrienoic acid (11,12-EET), 5Z,8Z,11Z,14Z-Eicosatetraenedioic acid (20cooh AA), 4Z,7Z,10Z,13Z,16Z,19Z-docosahexaenoic acid (DHA), 5Z,8Z,11Z,14Z,17Z-eicosapentaenoic acid (EPA), 5Z,8Z,11Z,14Z-eicosatetraenoic acid (Arachidonic Acid), 16-hydroxy-4Z,7Z,10Z,13Z,17E,19Z-docosahexaenoic acid (16-HDoHE), 15-hydroxy-8Z,11Z,13E-eicosatrienoic acid (15-HETrE), 15-hydroxy-5Z,8Z,11Z,13E-eicosatetraenoic acid (15-HETE), 5-hydroxy-6E,8Z,11Z,14Z-eicosatetraenoic acid (5-HETE), 8-hydroxy-9E,11Z,14Z-eicosatrienoic acid (8-HETrE), 11-hydroxy-5Z,8Z,11E,14Z-eicosatetraenoic acid (11-HETE), 7,10,13,16-docosatetraenoic acid (Adrenic Acid), 5-hydroxy-6E,8Z,11Z-eicosatrienoic acid (5-HETrE), 9-hydroxy-10E,12Z,15Z-octadecatrienoic acid (9-HOTrE), 14-hydroxy-4Z,7Z,10Z,12E,16Z,19Z-docosahexaenoic acid (14-HDoHE), 12-hydroxy-5Z,8Z,10E,14Z,17Z-eicosapentaenoic acid (12-HEPE), 12-hydroxy-5Z,8Z,10E,14Z-eicosatetraenoic acid (12-HETE), 4-hydroxy-5E,7Z,10Z,13Z,16Z,19Z-docosahexaenoic acid (4-HDoHE).

To test for collinearities between the variables and to identify characteristic patterns within the dataset, we constructed a correlation matrix (Fig. 1). As can be seen, a high degree of correlation was found between specific hydroxylated fatty acids including 5-, 8-, 11-, and 15-HETE. A notable exception was 12-HETE, which did not fall into this general group but correlated with some hydroxylated w-3 fatty acids including 12-HEPE and 14-HDoHE. Additionally, most dihydroxylated fatty acids also showed significant correlation, as well as their pre-cursor epoxides that are derived from the cytochrome P450 pathway.

### Establishing a model to distinguish between MASLD and controls without MASLD

**Fig. 2:**
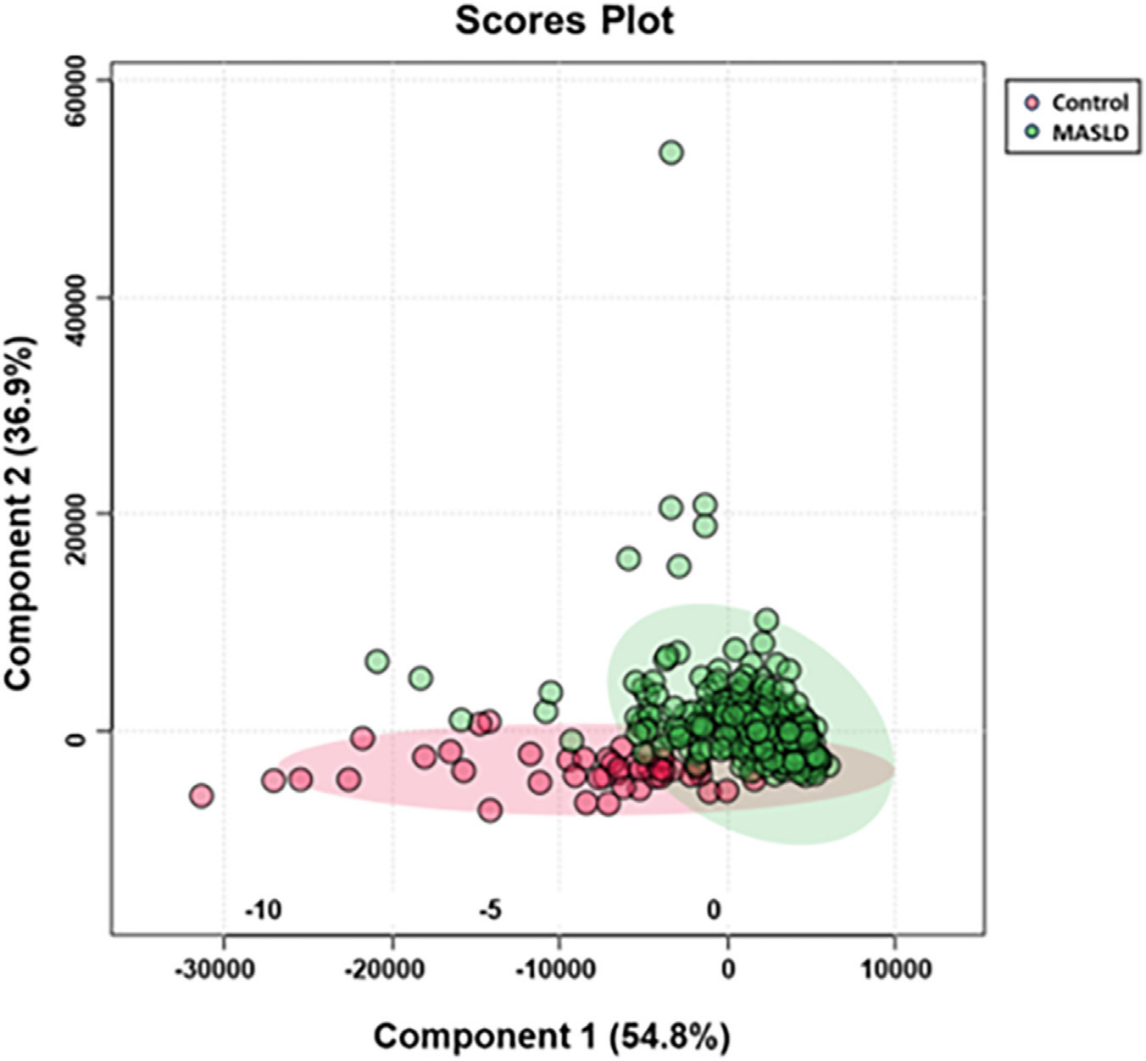
Partial least square analysis. Shown is the Scores Plot of PLS-DA for the 32 eicosanoids that were present in at least 80% of the samples, The analysis shows a clear separation between the control and MASLD groups. The partial overlap represents patients with very mild disease.

To examine if any of the changes across the eicosanoid profile are sufficient to discriminate between MASLD and controls, we performed a supervised partial least-square discriminant analysis (PLS-DA). The scores blot shown in Fig. 2 indicated that the changes in the plasma eicosanoid levels were sufficient to segregate MASLD from controls (Table 1).

**Fig. 3:**
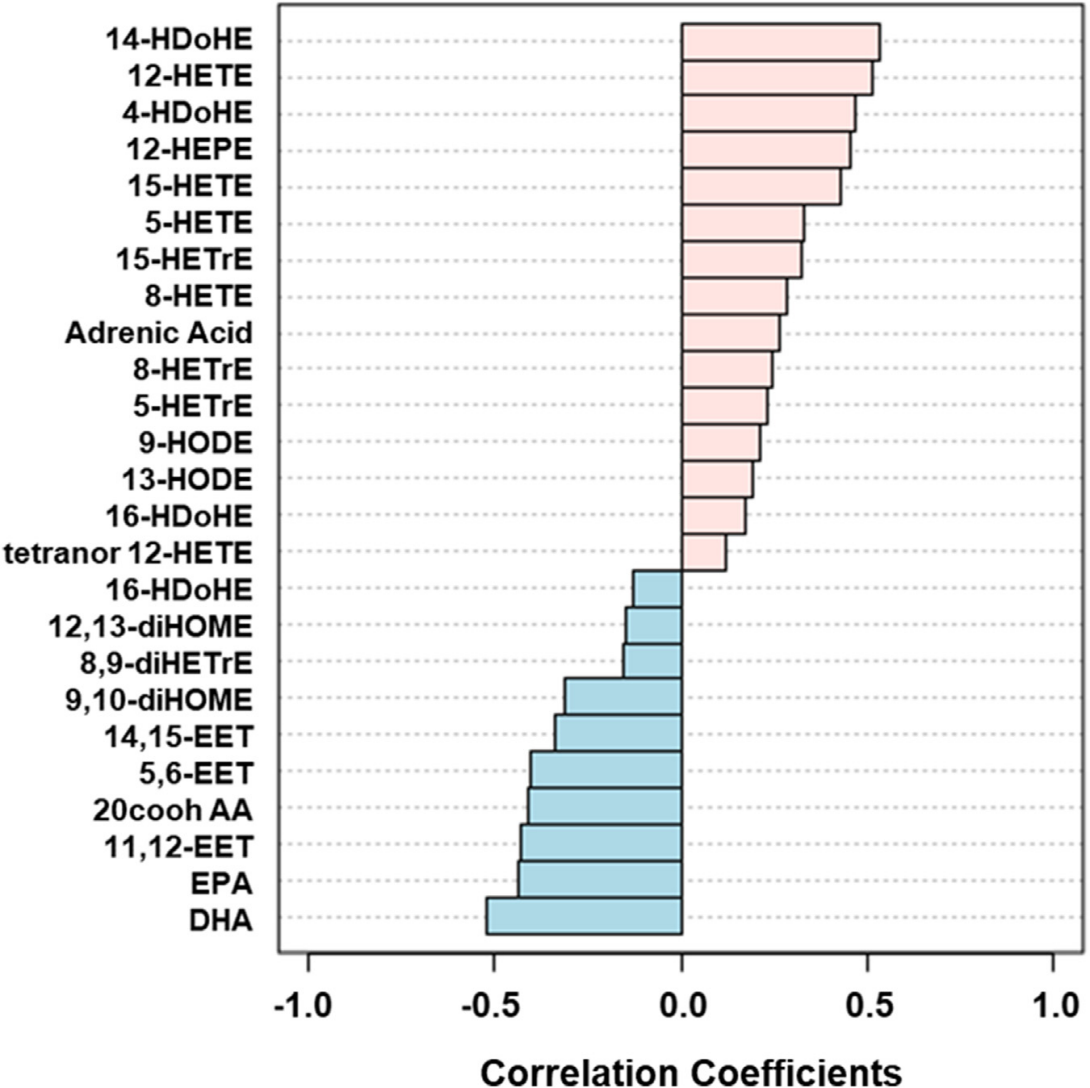
Eicosanoid changes in MASLD. Shown are the top 25 eicosanoids with the most robust changes in MASLD.

We then proceeded to examine whether any of the variables correlated with MASLD. Table 2 lists the top 25 variables that correlated with the disease. Figure 3 shows that 14-HDoHE and 12-HETE showed a strong positive correlation. In contrast, several of the epoxides including 5,6-EET and 11,12-EET showed a strong negative correlation, as did several of the fatty acids.

**Table 2:** Top 25 eicosanoids for the diagnosis of MASLD

Biomarker	AUROC	95% CI	*P* value	Log2 FC
14 HDoHE	0.95	0.92–0.97	0.16	−5.2
DHA	0.94	0.90–0.97	<0.001	1.4
12-HETE	0.93	0.90–0.97	0.15	−4.2
4 HDoHE	0.89	0.86–0.93	0.34	−9.0
12-HEPE	0.88	0.84–0.92	0.23	−4.6
EPA	0.86	0.81–0.92	<0.001	1.4
15-HETE	0.86	0.81–0.90	0.40	−5.6
11,12-EET	0.86	0.79–0.91	<0.001	1.1
20-COOH AA	0.84	0.79–0.87	<0.001	1.2
5,6-EET	0.83	0.78–0.87	<0.001	1.1
14,15-EET	0.78	0.73–0.84	0.54	−0.9
5-HETE	0.78	0.71–0.85	0.30	−6.9
15-HETrE	0.77	0.72–0.83	0.42	−5.7
9,10-diHOME	0.76	0.69–0.82	<0.001	0.8
8-HETE	0.74	0.66–0.80	0.41	−6.1
Adrenic Acid	0.72	0.66–0.78	<0.001	−0.9
8-HETrE	0.70	0.65–0.76	0.34	−6.0
5-HETrE	0.70	0.63–0.77	0.31	−5.8
9-HODE	0.68	0.60–0.74	0.28	−2.5
13-HODE	0.66	0.58–0.75	0.29	−2.1
16 HDoHE	0.64	0.58–0.73	0.35	−5.7
8,9-diHETrE	0.63	0.55–0.70	0.43	0.1
12,13-diHOME	0.62	0.55–0.70	0.08	0.3
5,6-diHETrE	0.61	0.54–0.67	0.55	−2.8
tetranor 12-HETE	0.60	0.54–0.67	0.12	−0.9

FC, fold change.

**Fig. 4:**
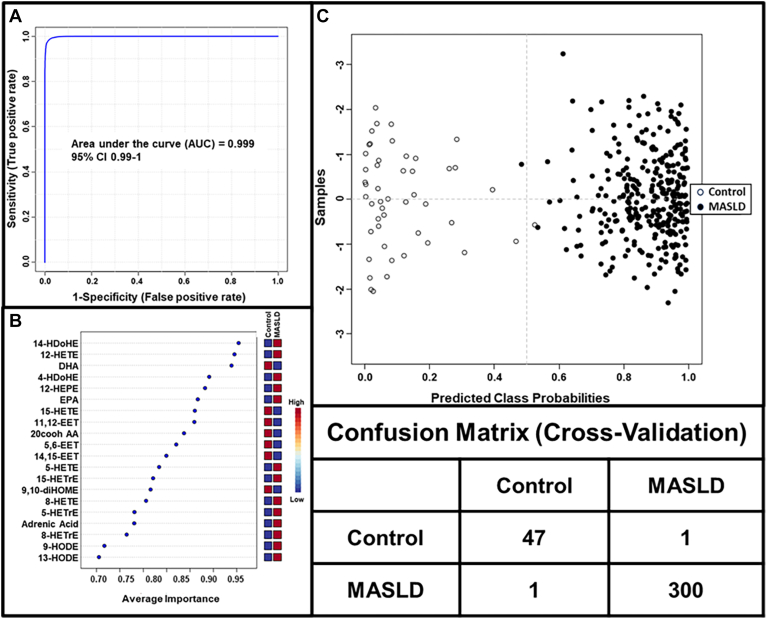
Biomarker analysis for MASLD. For analysis, all 32 eicosanoids are present in at least 80% of plasma. A: Multivariate AUROC analysis of 32 eicosanoids using Random Forests as the classification method. B: Average importance of the top 20 eicosanoids using Univariate AUROC as a ranking method. C: Predicted class probability and chart for each corresponding confusion matrix.

The performance of the features selected by PLS-DA was tested by AUROC analysis using the Random Forest approach (Fig. 4). As can be seen, using all 32 variables, the performance is almost perfect at 0.999. Using the confusion matrix, only one control and one MASLD patient were misclassified. The top 20 eicosanoids with the best discriminatory power to distinguish MASLD from controls were identified.

### Selection of a final eicosanoid panel for MASLD and clinical performance of the panel

**Fig. 5:**
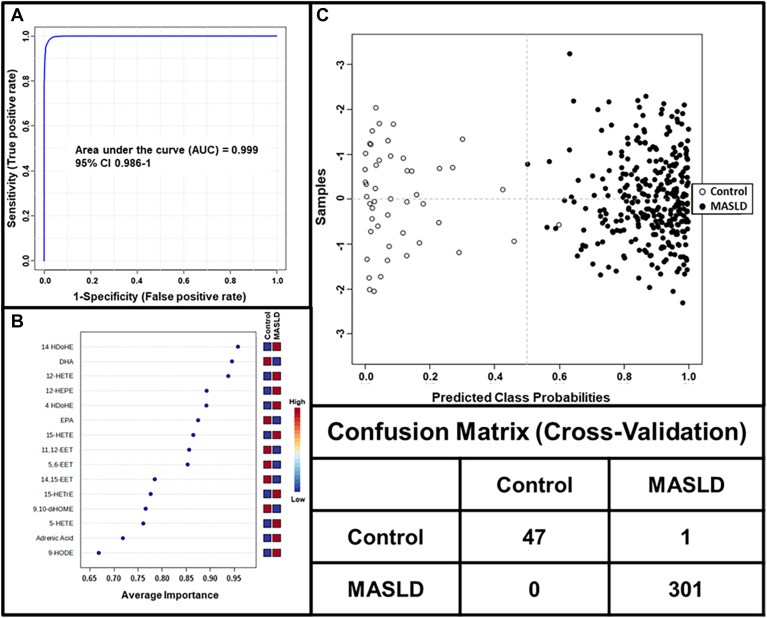
Final model for the diagnosis of MASLD. Fifteen eicosanoids were selected to optimally establish a final panel that best distinguishes between the control group and MASLD. A: Multivariate AUROC analysis of 15 eicosanoid panel using Random Forests as the classification method. B: Average importance using Univariate AUROC as ranking method. C: Predicted class probability chart for each corresponding confusion matrix.

Several of the detectable eicosanoids were present in plasma at low levels and showed considerable variabilities. Moreover, a number of eicosanoids displayed high degrees of collinearity and thus were not further considered as independent variables. Thus, the top 15 eicosanoids that best distinguished between controls and MASLD were used to further develop an “Eicosanoid Lipidomics Panel” and a model to diagnose MASLD (Table 3). We assessed their individual diagnostic test performances using AUROC (Fig. 5). The panel demonstrated an AUROC of 0.999 with a 95% confidence interval CI of 0.986–1.0. The predicted class probabilities and the confusion matrix show that out of 48 controls and 301 MASLD patients, only one control was misclassified. The reliability of the model was further tested by analyzing the performance of each of the 15 analytes that comprise the panel. As shown in Supplemental Figure S1, the levels of many individual analytes were significantly different between controls and MASLD.

**Table 3:** Selected final 15 eicosanoid lipidomics panel for the diagnosis of MASLD

Biomarker	AUROC	95% CI	*P* value	Log2 FC
14 HDoHE	0.95	0.92–0.97	0.16	−5.2
DHA	0.94	0.90–0.97	<0.001	1.4
12-HETE	0.93	0.90–0.97	0.15	−4.2
4 HDoHE	0.89	0.86–0.93	0.36	−9.0
12-HEPE	0.88	0.84–0.92	0.23	−4.6
EPA	0.86	0.81–0.92	<0.001	1.4
15-HETE	0.86	0.81–0.90	0.40	−5.6
11,12-EET	0.86	0.79–0.91	<0.001	1.1
5,6-EET	0.83	0.78–0.87	<0.001	1.1
14,15-EET	0.78	0.73–0.84	0.54	−0.90
5-HETE	0.78	0.71–0.85	0.30	−6.9
15-HETrE	0.77	0.72–0.83	0.42	−5.7
9,10-diHOME	0.76	0.69–0.82	<0.001	0.8
Adrenic Acid	0.72	0.66–0.78	<0.001	−0.9
9-HODE	0.68	0.60–0.74	0.28	−2.5

FC, fold change.

### Composite score for diagnostic application

To be useful for clinical applications, we provide a composite scoring algorithm that can be used to distinguish between control and MASLD. To accomplish this, we first established the cutoff values for each of the eicosanoids in the panel that distinguish between controls and MASLD. Because the values of some of the metabolites, especially the fatty acids, are orders of magnitudes higher than those of the eicosanoid metabolites, it is impractical to use averages as a composite value. Small percentage differences in the plasma fatty acids that are 3 to 4 orders of magnitude more abundant than eicosanoids would skew the classification disproportionally. Moreover, some of the eicosanoids are increased in MASLD and some are increased in the controls. For these reasons, we opted for a binary system to calculate a score that best separates controls from MASLD.

To create the composite score, we first established the absolute cutoff values for each of the eicosanoids in the panel. We used the univariate ROC results, that provide the cutoff values that best separate controls from MASLD (Table 4). We applied these values across the selected eicosanoid panel to generate the binary value of 0 or 1. For each sample, if the levels of any of the eicosanoids include 4-HDoHE, 5-HETE, 9-HODE, 12-HEPE, 12-HETE, 14-HDoHE, 15-HETE. 15-HETrE, or adrenic acid were above the cutoff, their score was 1. Conversely, some of the eicosanoid metabolites decrease in MASLD. If any of the eicosanoids, including 9,10-diHOME, 11,12-EET, 5,6-EET, 14,15-EET, DHA, or EPA fall below the cutoff, their score is also 1. If any of the metabolites in any of the samples did not follow this pattern, they received a score of 0. Using this algorithm, we constructed a composite score to separate controls from MASLD. The composite score is the sum total of the binary value of each of the analytes that follows this framework. Using this approach, we concluded that a composite score of 6+ was optimal to diagnose MASLD (Table 4). This means that at least six metabolites of the 15 biomarkers must be present at concentrations indicative for MASLD to classify the sample as MASLD. Using the composite score of 6+, we can achieve a predictive accuracy of 99.4%. This stringency identifies all MASLD patients but misaligns two of the controls. Using a composite score of 5+ reduces the stringency and misaligns five controls to the MASLD group. Increasing the stringency to 7+ misclassifies 7 MASLD samples to the control category. Nonetheless, this “MASLD LIPIDOMICS SCORE” scoring system can be dynamically tuned in future studies by either adjusting the individual metabolite cutoff values and/or adjusting the composite scoring.

**Table 4:** Eicosanoid lipidomics panel scoring chart for MASLD

Biomarker	Cutoff (pmol/ml)	Number of samples classified as MASLD		
		Control (N = 48)	MASLD (N = 301)	Accuracy
Increased in MASLD		**Score 5+**		
4-HDoHE	0.145	5	301	98.6%
5-HETE	1.78	**Score 6+**		
9-HODE	23.4	2	301	99.4%
12-HEPE	0.885	**Score 7+**		
12-HETE	3.32	2	294	97.4%
14-HDoHE	0.93	**Score 8+**		
15-HETE	0.665	0	292	97.4%
15-HETrE	0.235			
Adrenic Acid	1420			
Decreased in MASLD				
9,10-diHOME	3.16			
11,12-EET	0.065			
5,6-EET	0.035			
14,15-EET	0.365			
DHA	7260			
EPA	5160			

### Validation study

To validate the findings, a separate cohort consisting of 122 MASLD samples and 30 control samples was analyzed. The characteristics by MASLD status is shown in Supplemental Table S2 and the mean eicosanoid values in the previously described fifteen analyte panels are listed in Supplemental Table S3. Supplemental Figure S2 shows the Validation Study box plots for the 15 eicosanoids that were selected in the original study The eicosanoid panel scoring chart for MASLD in the Validation Study is shown in Table 5. In the Validation Study, we used the same cutoff values that were determined in the original study. Using these values and the MASLD LIPIDOMICS SCORE of 6+, there were three false positives among the controls and 1 false negative among the MASLD cohort.

**Table 5:** Eicosanoid panel scoring chart for MASLD in validation study

Biomarker	Cutoff (pmol/ml)	Number of samples classified as MASLD		
		Control (N = 30)	MASLD (N = 122)	Accuracy
Increased in MASLD		**Score 5+**		
4-HDoHE	0.145	11	122	92.8%
5-HETE	1.78	**Score 6+**		
9-HODE	23.4	3	121	97.4%
12-HEPE	0.885	**Score 7+**		
12-HETE	3.32	1	115	94.7%
14-HDoHE	0.93	**Score 8+**		
15-HETE	0.665	0	98	84.2%
15-HETrE	0.235			
Adrenic Acid	1420			
Decreased in MASLD				
9,10-diHOME	3.16			
11,12-EET	0.065			
5,6-EET	0.035			
14,15-EET	0.365			
DHA	7260			
EPA	5160			

The same cutoff values were used for the validation study as for the original study.

## Discussion

Approximately 75% of the general adult population is overweight or obese, the principal risk factor for MASLD (1, 2). MASLD, however, has a prevalence of 23%–32%. In a previous report, we identified a panel of three lipid metabolites including 11,12-diHETrE, dhk PGD2, and 20-COOH AA that accurately predicted MASLD and was able to discriminate between steatosis and steatohepatitis (36). By design, this was a small pilot study with 10 controls, 10 patients with steatosis, and nine patients with biopsy-proven MASH. In another pilot study, we also observed a correlation of certain eicosanoids with fibrosis (37). Several other independent metabolomics and lipidomics studies were performed to identify biomarkers for MASLD and MASH. The findings of these studies are summarized in a comprehensive review (38). We now carried out a much bigger follow-up study on 301 MASLD patients and 48 controls as well as a validation study. The patient population represented the entire spectrum of disease progression from simple steatosis with no inflammation to end-stage liver disease. The study aimed to identify a panel of bioactive lipids that can accurately identify MASLD at any stage; mild, moderate, or severe, with or without inflammation and fibrosis, and with or without underlying obesity. In all, 77 eicosanoid metabolites were detectable in at least one of the plasma samples. For a metabolite to be of clinical relevance as a biomarker, it must be present consistently and in measurable amounts. We concluded that only metabolites that were present in 80% of all control and patient plasma samples should be included in further analysis. The application of such a stringent algorithm reduced the panel of potential biomarkers to 32 eicosanoid metabolites.

Eicosanoids are signaling molecules that can be formed by enzymatic or non-enzymatic oxidation of arachidonic acid or similar elongated unsaturated fatty acids (29). The biosynthesis of eicosanoids via the enzymatic pathways includes the cyclooxygenase (COX), lipoxygenases (LOX), and cytochrome P450 pathways. To avoid overfitting the statistical model in the biomarker search, we aimed to prevent an over-representation of a specific pathway. We recognized that metabolites of a specific pathway are all increased or decreased if the underlying biosynthetic pathway is up- or downregulated. Thus, multiple eicosanoid metabolites derived from a single pathway should not be considered independent variables. To address this issue, we performed a correlation analysis (Fig. 1). As can be seen, several correlation clusters became apparent. For example, a high degree of correlation was found between some hydroxylated fatty acids. These presumably are derived from the LOX pathway. We reasoned that if the activity of the LOX pathway is changed in MASLD, then all metabolites within this pathway would change accordingly. Using all 32 metabolites in the AUROC analysis gave an excellent AUC of 0.999 (CI = 0.99–1.0). However, using the entire dataset, we noticed a partial overlap in the Scores Plot of the PLS-DA (Fig. 2). Some of the patients either presented with very mild disease or had progressed significantly to cirrhosis. At that stage, the liver loses much of the fat content, and the metabolic characteristics of the liver change significantly. In general, cirrhosis of the liver can readily be diagnosed, and the false negative classification should not pose any limitation in a clinical setting.

To improve the diagnostic performance, we selected a panel for the final model that consisted of 12 eicosanoids and three free fatty acids from the top 20 metabolites that were identified to be predictive for MASLD by univariant AUROC analysis. The panel was highly predictive for MASLD with an AUROC of 0.999 (95% CI = 0.986–1.0). As shown in Figure 5, of the 48 controls and of the 301 MASLD patients, only one control was incorrectly classified.

In summary, we identified a panel consisting of fifteen lipid metabolites (Table 4) that accurately predict MASLD. Furthermore, we developed a strategy to determine a MASLD LIPIDOMICS SCORE, which predicts the presence of MASLD with high accuracy based on lipidomics analysis of 50 ul of plasma. While we included a validation study herein, further optimization of the MASLD LIPIDOMICS SCORE and validation will still be required to establish the resulting MASLD LIPIDOMICS SCORE as a useful non-invasive “point-of-care” test to identify MASLD individuals requiring further evaluation for the presence of metabolic dysfunction-associated steatohepatitis (MASH). Ideally, this optimization would be carried out as part of a prospective study in a larger population chosen to balance all of the potentially confounding variables including BMI, diabetes and cardiovascular disease, gender, ethnicity, and age as well as liver enzymes and lipid levels, including an equal number of carefully matched controls. However, the potential of a MASLD LIPIDOMICS SCORE that can be used as a routine, non-invasive screening assay for fatty liver disease that will trigger physicians to carry out further patient evaluation and guide them to implement the appropriate treatment options will be a valuable addition to the arsenal of precision medicine.

## Data availability

Data will be shared upon written request to Edward Dennis at UCSD through email edennis@ucsd.edu, or to Oswald Quehenberger at UCSD through email oquehenberger@health.ucsd.edu.

## Supplemental data

This article contains supplemental data.

## Conflict of interest

The authors declare the following financial interests/personal relationships which may be considered as potential competing interests: EAD and RL are co-founders and hold equity in LipoNexus, Inc which has licensed technology from the University of California, San Diego. AS is a consultant and holds stock options in LipoNexus, Inc. Additionally, RL serves as a consultant to Aardvark Therapeutics, Altimmune, Arrowhead Pharmaceuticals, AstraZeneca, Cascade Pharmaceuticals, Eli Lilly, Gilead, Glympse bio, Inipharma, Intercept, Inventiva, Ionis, Janssen Inc., Lipidio, Madrigal, Neurobo, Novo Nordisk, Merck, Pfizer, Sagimet, 89 bio, Takeda, Terns Pharmaceuticals and Viking Therapeutics. He holds stock options in Sagimet Biosciences. His institution received research grants from Arrowhead Pharmaceuticals, AstraZeneca, Boehringer-Ingelheim, Bristol-Myers Squibb, Eli Lilly, Galectin Therapeutics, Gilead, Intercept, Hanmi, Intercept, Inventiva, Ionis, Janssen, Madrigal Pharmaceuticals, Merck, Novo Nordisk, Pfizer, Sonic Incytes and Terns Pharmaceuticals. Additionally, AS holds stock options in Genfit, Tiziana, Hemoshear, Rivus, Northsea, and Inversago. He has served as wa consultant to Gilead, Intercept, Boehringer Ingelhiem, Merck, Novo Nordisk, Eli Lilly, Madrigal, Alnylam, Hanmi, LG Chem, Takeda, Regeneron, Genentech, Siemens, Surrozen, Poxel, Path AI, Histoindex, Zydus, 89 Bio, Akero, and Salix. His institution has received grants from Gilead, Bristol Myers Squibb, Intercept, Novo Nordisk, Akero, Takeda, Merckw, Salix, Eli Lilly, Hanmi, Madrigal, Boehringer Ingelheim, and Pfizer. He received royalties from Elsevier and Uptodate.9.
