# Supplementary material for: Novel eicosanoid signature in plasma provides diagnostic for metabolic dysfunction-associated steatotic liver disease
Source: J Lipid Res. 2024 Sep 18;65(10):100647. doi: 10.1016/j.jlr.2024.100647 (PMC11526069; doi:10.1016/j.jlr.2024.100647)
Supplement: Supplemental data [file mmc1.pdf]

**SUPPLEMENTAL DATA**

# NOVEL EICOSANOID SIGNATURE IN PLASMA PROVIDES DIAGNOSTIC FOR METABOLIC DYSFUNCTION-ASSOCIATED STEATOTIC LIVER DISEASE

Oswald Quehenberger[1,*], Aaron M. Armando[1], Tiffany H. Cedeno[1], Rohit Loomba[2], Arun J. Sanyal[3], and Edward A. Dennis[1,4,*]

1. Department of Pharmacology, University of California San Diego, La Jolla, CA
2. MASLD Research Center, Division of Gastroenterology and Hepatology, Department of Medicine, University of California San Diego, La Jolla, CA
3. Division of Gastroenterology, Department of Internal Medicine, Virginia Commonwealth University School of Medicine, Richmond, VA
4. Department of Chemistry and Biochemistry, University of California San Diego, La Jolla, CA

*Address Correspondence to: Oswald Quehenberger oquehenberger@health.ucsd.edu or Edward A. Dennis edennis@ucsd.edu

**Table S1. Eicosanoids in the plasma of controls and MASLD patients.** Only analytes that were present in at least 80% of the samples are shown and used in the analysis, except for 14,15-EET and 5,6-EET that were present in 69% and 56%, respectively, above ND levels in the MASLD patients. These two EETs were present in all controls, but sharply decreased in MASLD and sometimes fell below the level of detection.

| **Analyte** | **Controls (*N* = 48)** | | **MASLD patients (*N* = 301)** | | **p *value*** |
|---|---|---|---|---|---|
| | Mean | SE | Mean | SE | |
| | pmol/ml | | pmol/ml | | |
| tetranor 12-HETE | 0.14 | 0.01 | 0.26 | 0.03 | 0.12 |
| 11-HETE | 0.42 | 0.02 | 33.4 | 16.5 | 0.43 |
| 16 HDoHE | 0.23 | 0.02 | 11.9 | 4.9 | 0.35 |
| 5-HETE | 1.76 | 0.12 | 214.0 | 80.7 | 0.30 |
| 4 HDoHE | 0.09 | 0.01 | 47.2 | 19.4 | 0.34 |
| 9-HOTrE | 0.24 | 0.02 | 1.58 | 0.49 | 0.28 |
| 5-HETrE | 0.08 | 0.01 | 4.20 | 1.61 | 0.31 |
| 15-HETE | 0.53 | 0.02 | 26.1 | 12.0 | 0.40 |
| 13-HODE | 19.2 | 1.7 | 84.2 | 24.4 | 0.29 |
| 15-HETrE | 0.18 | 0.01 | 9.15 | 4.42 | 0.42 |
| 8-HETE | 0.36 | 0.02 | 24.8 | 11.8 | 0.41 |
| 8-HETrE | 0.15 | 0.01 | 9.7 | 4.5 | 0.40 |
| 12-HETE | 2.22 | 0.34 | 42.1 | 10.9 | 0.15 |
| 12-HEPE | 0.15 | 0.02 | 3.58 | 1.13 | 0.23 |
| 14 HDoHE | 0.43 | 0.05 | 15.6 | 4.3 | 0.16 |
| 9-HODE | 21.1 | 1.8 | 115.4 | 34.8 | 0.28 |
| 18-HETE | 0.31 | 0.02 | 0.55 | 0.09 | 0.29 |
| 5,6-EET | 0.07 | 0.01 | 0.03 | 0.004 | <0.001 |
| 11,12-EET | 0.12 | 0.01 | 0.06 | 0.01 | <0.001 |
| 14,15-EET | 0.56 | 0.04 | 1.05 | 0.31 | 0.54 |
| 19,20-DiHDPA | 2.26 | 0.12 | 2.48 | 0.12 | 0.45 |
| 5,6-diHETrE | 0.48 | 0.03 | 0.58 | 0.07 | 0.55 |
| 8,9-diHETrE | 0.43 | 0.02 | 0.39 | 0.02 | 0.43 |
| 11,12-diHETrE | 0.79 | 0.04 | 0.98 | 0.04 | 0.06 |
| 14,15-diHETrE | 0.87 | 0.03 | 0.90 | 0.03 | 0.65 |
| 9,10-diHOME | 5.54 | 0.72 | 3.23 | 0.24 | <0.001 |
| 12,13-diHOME | 7.51 | 0.82 | 5.96 | 0.33 | 0.08 |
| Arachidonic Acid | 6626 | 347.9 | 7915 | 290.3 | 0.08 |
| Adrenic Acid | 1058 | 77.1 | 2013 | 82.4 | <0.001 |
| EPA | 9110 | 832.8 | 3417 | 187.1 | <0.001 |
| DHA | 12637 | 844.3 | 4801 | 153.5 | <0.001 |
| 20cooh AA | 23.1 | 2.6 | 10.0 | 0.9 | <0.001 |

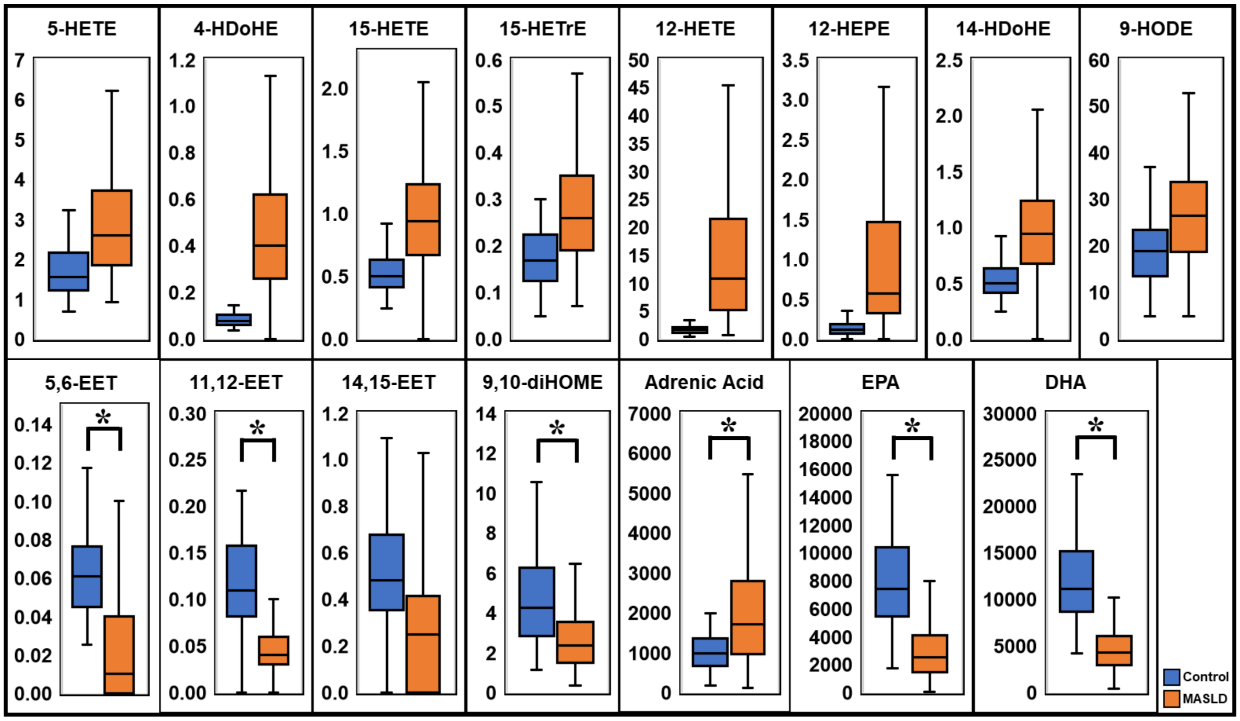


* p-value <0.001, p-values for all other analytes were not significant

**Figure S1. Box Plots of Selected Eicosanoid Panel.** Data for fifteen eicosanoids that were selected to optimally establish a panel that best distinguishes between controls and MASLD are shown as Box Plots. The data are expressed as pmol/ml plasma. Outliers with values more than 1.5 times the inter-quartile range above the 3rd quartile have been removed from the graphs for visualization, but all values were included in the calculation of the median.

**Table S2. Validation Study subject characteristics by MASLD status (N = 152)**

| | Mean (± SD) or *N* (%) | | | | *P** |
|---|---|---|---|---|---|
| | Control (*N* = 30) | | MASLD patients (*N* = 122) | | |
| Age (years) | 52.5 | (±14.7) | 51.8 | (±11.0) | 0.8 |
| Age | | | | | 0.9 |
| 18-34 | 5 | (17%) | 10 | (8%) | |
| 35-54 | 7 | (23%) | 52 | (43%) | |
| 55-74 | 18 | (60%) | 60 | (49%) | |
| Sex, male | 15 | (50%) | 36 | (30%) | 0.03 |
| Race | | | | | <0.001 |
| Non-Hispanic white | 9 | (30%) | 94 | (77%) | |
| Non-Hispanic black | 3 | (10%) | 2 | (2%) | |
| Hispanic | 15 | (50%) | 16 | (13%) | |
| Other | 3 | (10%) | 10 | (8%) | |
| BMI (kg/m$^2$) | 32.2 | (±4.6) | 34.9 | (±6.2) | 0.03 |
| BMI category | | | | | 0.10 |
| Underweight | 0 | (0%) | 0 | (0%) | |
| Normal | 0 | (0%) | 3 | (3% | |
| Overweight | 11 | (37%) | 23 | (19%) | |
| Obese | 19 | (63%) | 95 | (78%) | |
| Type 2 diabetes | 10 | (33%) | 58 | (48%) | 0.16 |
| Total Cholesterol (mg/dL | 178 | 38(±) | 194 | (±50) | 0.61 |
| Triglycerides (mg/dL) | 134 | 95(±) | 205 | (±286) | <0.001 |
| LDL (mg/dL) | 103 | 28(±) | 116 | (±44) | 0.13 |
| HDL(mg/dL) | 47 | 8(±) | 44 | (±13) | 0.13 |
| Bilirubin, total (mg/dL) | 0.4 | (±0.2) | 0.6 | (±0.3) | 0.005 |
| Aspartate aminotransferase, AST (U/L) | 19 | (±4) | 64 | (±36) | <0.001 |
| Alanine aminotransferase, ALT (U/L) | 18 | (±6) | 87 | (±51) | <0.001 |
| Alkaline phosphatase, ALP (U/L) | 77 | (±22) | 82 | (±26) | 0.33 |
| Fibrosis stage† | | | | | |
| 0. None | 30 | (100%) | 11 | (9%) | |
| 1a. Mild, zone 3 perisinusoidal | | | 9 | (7%) | |
| 1b. Moderate, zone 3, perisinusoidal | | | 13 | (11%) | |
| 1c. Portal/periportal only | | | 6 | (5%) | |
| 2. Zone 3 and periportal, any combination | | | 43 | (35%) | |
| 3. Bridging | | | 39 | (32%) | |
| 4. Cirrhosis | | | 1 | (1%) | |
| MASH stage† | | | | | |
| Not MASLD | 30 | (100%) | 0 | (0%) | |
| 0. MASLD, not MASH | 0 | (0%) | 12 | (10%) | |
| 1a. borderline MASH, zone 3 pattern | 0 | (0%) | 8 | (7%) | |
| 1b. borderline MASH, zone 1 periportal pattern | 0 | (0%) | 1 | (<1%) | |
| 2. Definite MASH | 0 | (0%) | 101 | (83%) | |
| Time difference between lab exam and biopsy (day)‡ | | | 45 | (±118) | |

*$P$-value from student t-test for continuous variables and Fisher's exact test for categorical variables.

† Biopsy not done for controls.

‡ Date of lab exam – date of biopsy.

**Table S3. Validation Study Eicosanoids in the plasma of controls and MASLD patients.** Only analytes that were in the selected fifteen analyte panel are shown for the Validation Study.

| Analyte | Controls ($N$ = 30) | | MASLD patients ($N$ = 122) | | p *value* |
|---|---|---|---|---|---|
| | Mean | SE | Mean | SE | |
| | pmol/ml | | pmol/ml | | |
| 5-HETE | 0.85 | 0.09 | 187.59 | 111.1 | 0.41 |
| 4 HDoHE | 0.92 | 0.59 | 39.15 | 24.0 | 0.43 |
| 15-HETE | 0.62 | 0.09 | 11.22 | 6.2 | 0.40 |
| 15-HETrE | 0.22 | 0.06 | 6.68 | 4.2 | 0.45 |
| 12-HETE | 3.73 | 0.87 | 21.70 | 7.3 | 0.23 |
| 12-HEPE | 0.33 | 0.11 | 1.57 | 0.5 | 0.21 |
| 14 HDoHE | 1.26 | 0.34 | 9.28 | 3.5 | 0.26 |
| 9-HODE | 17.90 | 5.55 | 58.14 | 24.5 | 0.42 |
| 5,6-EET | 0.18 | 0.05 | 0.01 | 0.001 | <0.001 |
| 11,12-EET | 0.10 | 0.05 | 0.05 | 0.02 | 0.24 |
| 14,15-EET | 1.89 | 0.25 | 0.19 | 0.1 | <0.001 |
| 9,10-diHOME | 5.34 | 0.68 | 4.36 | 0.6 | 0.43 |
| Adrenic Acid | 1374 | 216 | 900 | 98 | 0.03 |
| EPA | 7935 | 1189 | 4713 | 841 | 0.44 |
| DHA | 25689 | 3489 | 4645 | 298 | <0.001 |

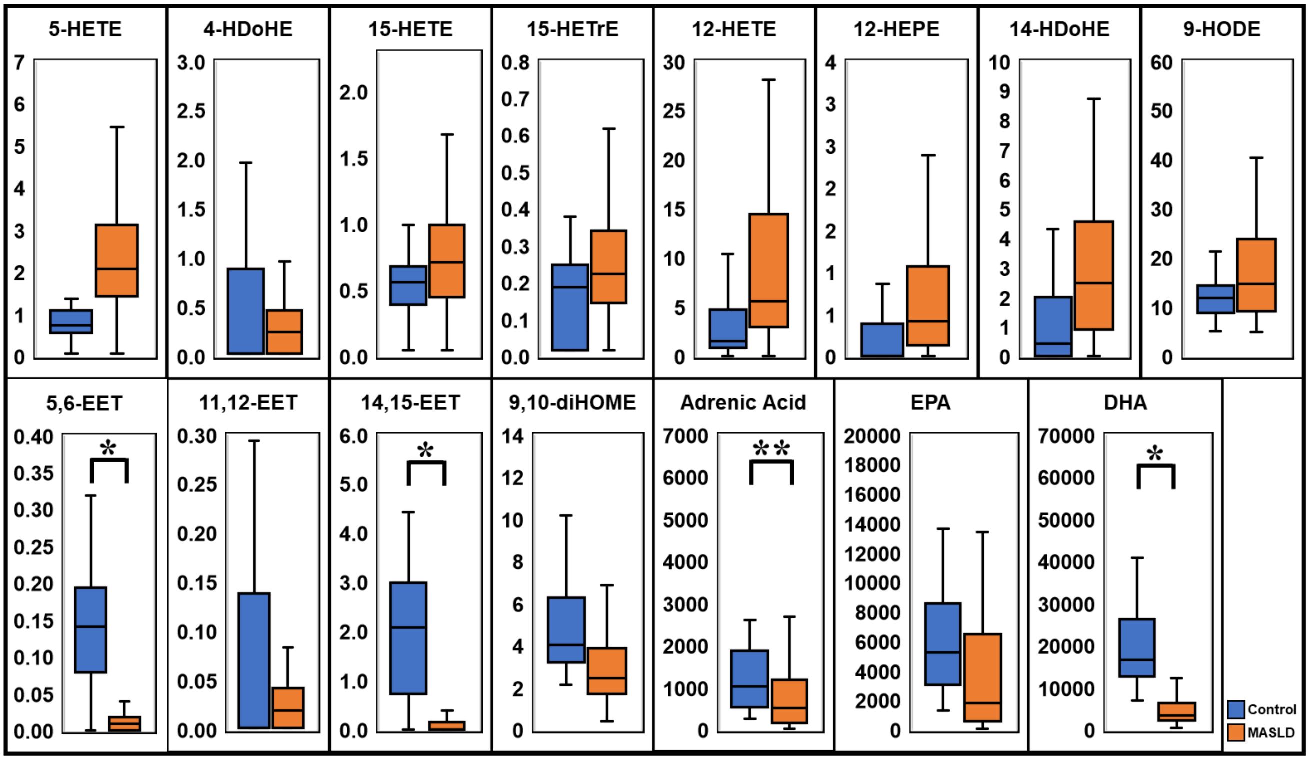


* p-value <0.001

** p-value <0.05

p-values for all other analytes were not significant

**Figure S2. Box Plots of Selected Eicosanoid Panel for Validation Study**. Data for the fifteen eicosanoids selected in the original study are shown for the Validation Study. The data are expressed as pmol/ml plasma. Outliers with values more than 1.5 times the inter-quartile range above the 3rd quartile have been removed from the graphs for visualization, but all values were included in the calculation of the median.
